# Effect of Preoperative Tamsulosin on Postoperative Urinary Retention Prevention After Sling Placement: A Randomized Controlled Trial

**DOI:** 10.1007/s00192-025-06120-2

**Published:** 2025-04-12

**Authors:** Angela Leffelman, Henry Chill, Claudia Paya-Ten, Alireza Hadizadeh, Jungeun Lee, Cecilia Chang, Ghazaleh Rostaminia

**Affiliations:** 1 Division of Urogynecology, University of Chicago Pritzker School of Medicine, Northshore University Healthsystem, 9650 Gross Point Road, Suite 3900, Skokie, IL 60076 USA; 2 Department of Obstetrics and Gynecology, Faculty of Medicine, Hadassah Medical Center, Hebrew University of Jerusalem, Jerusalem, Israel; 3 Northshore University Healthsystem Research Institute, Evanston, IL USA

**Keywords:** Mid-urethral sling, Postoperative urinary retention, Tamsulosin

## Abstract

**Introduction and Hypothesis:**

The objective of this study was to evaluate whether preoperative administration of tamsulosin would decrease the frequency of postoperative urinary retention (POUR) after mid-urethral sling (MUS) placement

**Methods:**

This was a prospective randomized, double-blinded, placebo-controlled trial of patients with SUI who underwent elective MUS placement at a single institution. Patients scheduled to undergo MUS placement consented, were enrolled, and were randomized to receive either a single tablet of tamsulosin 0.4 mg or placebo in the preoperative holding area on the day of their surgery. We then evaluated the rate of POUR after administration of tamsulosin compared with placebo using appropriate statistical methods. Sample size was calculated to include 160 patients.

**Results:**

A total of 161 patients (81 placebo, 80 tamsulosin) were analyzed. The incidence of POUR was similar between the tamsulosin and placebo groups (17.7% vs 19.8%, *p* = 0.7420). Secondary outcomes, including unplanned admissions, urinary-tract infections (UTIs), and hypotension, did not differ significantly between groups. A subgroup analysis of patients undergoing MUS without concomitant prolapse surgery suggested a trend toward lower POUR rates in the tamsulosin group (7.5% vs 16.7%, *p* = 0.142), although this was not statistically significant.

**Conclusion:**

These results suggest that single-dose preoperative tamsulosin might not have an effect on postoperative urinary retention after MUS placement, with or without concomitant reconstructive pelvic surgery.

## Introduction

Postoperative urinary retention (POUR) has been defined as the inability to void despite having fluid in the bladder during the postoperative period [1, 2]. Urinary retention after pelvic reconstructive surgery requiring indwelling-catheter usage or self-catheterization occurs in approximately 30–60% of patients postoperatively [2–4]. The retro-fill voiding trial is the most common method for diagnosing POUR. The bladder is filled with 300 ml of sterile water, and after catheter removal, the voided volume is compared with the PVR. Passing is typically defined as voiding at least two-thirds of the filled volume or at least 200 ml with a voided volume greater than the PVR. Failure results in a POUR diagnosis, requiring indwelling-catheter placement to prevent complications such as bladder overdistention, pain, and UTIs [5].

Tamsulosin is an alpha-adrenergic receptor blocker, which is thought to increase smooth-muscle relaxation and improve urinary flow [6]. Current literature has been primarily focused on the effect of tamsulosin in men with benign prostatic hyperplasia. Although extensively studied in men with benign prostatic hyperplasia, its primary mechanism of action involves relaxation of smooth muscle in the prostate and urethra, which may explain why its effect on postoperative urinary retention (POUR) has been more pronounced in male populations than in female cohorts. However, tamsulosin may be beneficial in women as well, with limited studies on postoperative urinary retention [6]. Chapman et al. published a randomized controlled trial evaluating postoperative urinary retention after female pelvic reconstructive surgery [7]. These patients underwent 10 days of tamsulosin (3 days preoperatively and 7 days postoperatively) and were found to have a 65% decrease in the urinary retention rate, from 25.8% to 8.8% [7]. Several other studies utilizing different regimens of treatment with tamsulosin support these findings, showing that tamsulosin may decrease POUR postoperatively in men and women [8–11].

Postoperative urinary retention is common after pelvic reconstructive surgery with mid-urethral sling (MUS) placement and is extremely bothersome to patients. Tamsulosin reaches its peak plasma concentration within 4 to 5 h in a fasting patient and has an elimination half-life of 9 to 13 h, providing a bioactive duration of approximately 24 h. Studies have shown that initial smooth muscle relaxation at the bladder neck occurs within 4 to 8 h after ingestion [12–14]. Given the simplicity of this single-dose regimen and its potential for higher patient adherence, we designed and conducted this study to evaluate its efficacy in preventing POUR following MUS placement. To our knowledge, no study to date has evaluated preoperative administration of single-dose tamsulosin for postoperative urinary retention after MUS placement in a randomized placebo-controlled trial.

The primary objective of this study was to evaluate whether preoperative administration of tamsulosin would decrease the rate of postoperative urinary retention after MUS placement with or without concomitant prolapse surgery compared with placebo. The secondary objective of this study was to evaluate for any differences in hypotension, unplanned admission, postoperative UTI, or unplanned 30-day health care encounters after preoperative tamsulosin compared with placebo.

## Materials and Methods

This was a prospective, randomized, double-blinded, placebo-controlled trial conducted at a single academic institution in the Midwest, USA, involving patients undergoing elective MUS placement, with or without concomitant prolapse surgery. This study underwent full-board review, was approved (IRB# EH22 - 470), and was registered with clinicaltrials.gov (ID# NCT05753670) prior to enrollment.

Women presenting to our tertiary urogynecology clinic with stress urinary incontinence who decided to proceed with surgical treatment via retropubic MUS placement were invited to participate in this study. Written consent was obtained.

Patients who underwent a MUS placement were enrolled (after the previously described informed consent discussion) between July 2023 and May 2024 to receive either a single tablet of tamsulosin 0.4 mg or a placebo in the preoperative holding area on the day of their scheduled surgery. Our exclusion criteria included subjects aged less than 18 years, planned combined cases with colorectal surgery, general surgery, or gynecologic oncology, history of prior sling placement, known history of urinary retention, or contraindication to tamsulosin.

We employed a centralized randomization process managed by an independent statistician not involved in patient recruitment or data collection. Participants were randomly assigned to either the tamsulosin or placebo group using a computer-generated sequence, ensuring that the allocation was unpredictable and concealed from the enrolling clinicians, any staff involved in medication administration, and the subjects themselves. This approach prevented selection bias by ensuring that the treatment assignment remains unknown until the point of allocation and the allocation was revealed only after the study was completed. Simple randomization (1:1 randomization) took place with a computer-generated random number list. Subjects were randomized to one of the two groups (preoperative tamsulosin 0.4 mg tablet versus placebo). The clinicians, preoperative and postoperative nursing staff, and the patients were blinded to the trial group and medication.

To maintain blinding, both the tamsulosin and placebo capsules were identical in appearance, taste, and packaging. Neither the participants nor the health care providers administering the interventions were aware of the group assignments. Outcome assessors and data analysts were also blinded to the allocation throughout the study period. Blinding was maintained by labeling the study medications with unique participant codes corresponding to the randomization sequence, without revealing the actual treatment. In the event of a medical emergency necessitating knowledge of the assigned treatment, a predefined code-break procedure was in place to unblind the specific participant without compromising the blinding of the remaining study personnel.

After surgery, patients underwent a retro-fill voiding trial in the recovery area. As described in prior studies, 4–6 h post-surgery, the bladder was back-filled with a set amount of sterile water (300 ml), the catheter is removed, the patient was permitted to void and the voided volume is compared with a post-void residual (PVR) volume. To assess the PVR, ultrasound bladder scanning is performed by placing a handheld transducer on the suprapubic area after the patient has attempted to void, and the device uses sound waves to estimate the bladder volume and calculate the PVR [15, 16]. The patient was considered to have failed the voiding trial if the voided volume was less than one-third of the total bladder volume or the PVR was ≥ 200 ml [17]. Upon failing the voiding trial, the patient was discharged home with a Foley catheter. We then compared the rate of postoperative urinary retention after preoperative administration of tamsulosin compared with placebo.

Prior to the study, the evaluated rate of postoperative urinary retention at our institution based on chart review was 25%. Published data discussed above have described a postoperative urinary retention rate decrease of 65–88% after perioperative tamsulosin administration [10–14]. Based on a sample-size calculation to achieve 80% power, to evaluate for a 65% decrease in our urinary retention rate (from 25% to approximately 8.8%) and 10% attrition rate, we needed to recruit a total of 160 subjects (80 per group).

Data were analyzed using SAS 9.4 (SAS Inc., Cary, NC, USA). Demographic and clinical characteristics of the groups was compared using Student’s *t* test (parametric) or Mann–Whitney *U* test (nonparametric) for continuous variables and Chi-squared test or Fisher’s exact test for categorical variables. Statistical significance was defined at *p* value < 0.05.

## Results

**Fig. 1:**
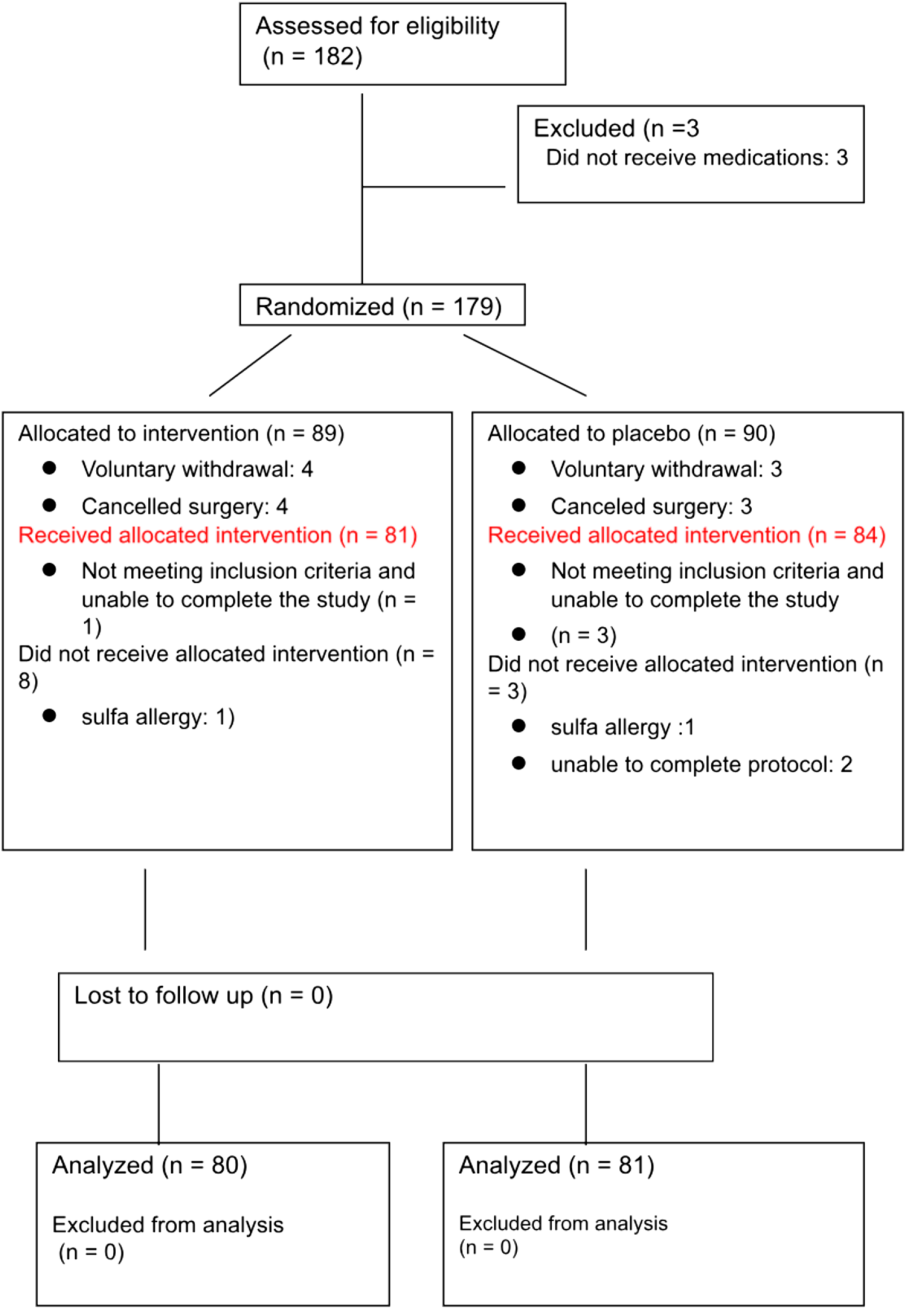
Enrollment diagram

We enrolled 182 subjects between 1 July 2023 and 31 May 2024. Two subjects developed a new allergy between enrollment and participation, which excluded their ability to receive the trial medication, 3 subjects did not receive the trial drug despite enrollment, 7 subjects voluntarily withdrew from the study, 7 subjects cancelled their surgery after enrollment, and 2 subjects had surgical complications that prevented them from completing the study protocol. We completed and performed our data analysis on 161 subjects (81 in the placebo group and 80 in the tamsulosin group; Fig. 1). Demographic and preoperative medical-history variables were similar in the two groups, with the exception of a higher rate of anxiety and depression in the tamsulosin group (21.8% vs 8.8%, *p* = 0.022; Table 1). Mean age in this cohort was 56.5 1 3.3, BMI was 28.4 ± 5.7, 57% of subjects were postmenopausal, and the majority of patients identified as white (73.9%).

**Table 1:** Patient characteristics

	Total (*N* = 161)		Placebo (*N* = 81)		Tamsulosin (*N* = 80)		*p* value
Demographics							
Age, mean ± SD	161	56.53 ± 13.33	81	56.35 ± 14.31	80	56.73 ± 12.35	0.8574
Parity, median (range)	154	2 (0–6)	79	2 (0–5)	75	2 (0–6)	0.8207
Race, *n* (%)							
American Indian/Alaska native	1 (0.62)		1 (1.23)		0 (0.00)		0.5877
Asian	8 (4.97)		4 (4.94)		4 (5.00)		
Black or African American	3 (1.86)		2 (2.47)		1 (1.25)		
White	119 (73.91)		56 (69.14)		63 (78.75)		
Other	28 (17.39)		16 (19.75)		12 (15.00)		
Unknown	2 (1.24)		2 (2.47)		0 (0.00)		
Ethnicity, *n* (%)							
Non-Hispanic	135 (83.85)		70 (86.42)		65 (81.25)		0.3728
Hispanic	26 (16.15)		11 (13.58)		15 (18.75)		
Smoking status, *n* (%)							
Nonsmoker	114 (72.15)		56 (70.00)		58 (74.36)		0.6395
Prior smoker	35 (22.15)		18 (22.50)		17 (21.79)		
Current smoker	9 (5.70)		6 (7.50)		3 (3.85)		
Menopausal status, *n* (%)							
Pre	66 (41.25)		35 (43.75)		31 (38.75)		0.5206
Post	94 (58.76)		45 (56.25)		49 (61.25)		
BMI, mean ± SD	161	28.35 ± 5.71	81	27.82 ± 5.42	80	28.88 ± 5.97	0.2404
Comorbid conditions, *n* (%)							
Diabetes mellitus	15 (9.49)		7 (8.75)		8 (10.26)		0.7467
Hypertension	42 (26.58)		25 (31.25)		17 (21.79)		0.1786
Cardiovascular diseases	13 (8.23)		8 (10.00)		5 (6.41)		0.4117
Respiratory diseases	27 (17.09)		15 (18.75)		12 (15.38)		0.5742
Obstructive sleep apnea	11 (7.05)		4 (5.13)		7 (8.97)		0.3481
Chronic pain	2 (1.27)		2 (2.50)		0 (0.00)		0.4969
Depression/anxiety	24 (15.19)		7 (8.75)		17 (21.79)		0.0224
Surgical history, *n* (%)							
Prior hysterectomy	14 (8.86)		6 (7.50)		8 (10.26)		0.5422
Prior abdominal surgery	48 (30.38)		20 (25.00)		28 (35.90)		0.1365
Prior prolapse surgery	1 (0.63)		1 (1.25)		0 (0.00)		1.0000

*BMI* body mass index, *SD* standard deviation

On preoperative urinary symptoms or prolapse stage, the tamsulosin group had a slightly higher total volume capacity (361.2 ml vs 330.1 ml, *p* = 0.0409); however, this difference did not appear to be clinically significant (Table 2). The tamsulosin group was also more likely to report nocturia (1.3 episodes per night vs 0.8 episodes per night, *p* = 0.0269; Table 2). Preoperative testing information is presented in Table 2.

**Table 2:** Pelvic Floor Disability Index (PFDI- 20), Pelvic-Organ Prolapse Quantification (POP-Q), and cystometrogram/urodynamics (CMG/UDS) measurements

	Total (*N* = 161)		Placebo (*N* = 81)		Tamsulosin (*N* = 80)		*p* value
PFDI- 20 urinary frequency/void interval per hour, mean ± SD	145	2.56 ± 1.51	74	2.32 ± 1.19	71	2.80 ± 1.75	0.0581
PFDI- 20 nocturia per night, mean ± SD	144	1.42 ± 1.11	73	1.29 ± 0.99	71	1.56 ± 1.20	0.1356
PFDI- 20 UUI episodes per day, mean ± SD	142	1.04 ± 1.37	72	0.79 ± 1.22	70	1.30 ± 1.48	0.0269
PFDI- 20 SUI episodes per day, mean ± SD	142	2.65 ± 2.36	72	2.64 ± 2.12	70	2.66 ± 2.59	0.9579
Prolapse grade, *n* (%)							
0: no prolapse	61 (42.36)		35 (47.3)		26 (37.14)		0.7103
1	15 (10.42)		6 (8.11)		9 (12.86)		
2	37 (25.69)		18 (924.32)		19 (27.14)		
3	22 (15.28)		10 (913.51)		12 (17.14)		
4	9 (6.25)		5 (96.76)		4 (5.71)		
POP-Q, mean ± SD							
Aa	41	0.51 ± 1.68	15	0.33 ± 1.42	26	0.62 ± 1.83	0.6114
Ba	56	0.73 ± 1.73	25	0.78 ± 1.72	31	0.69 ± 1.77	0.8549
C	48	− 2.57 ± 3.70	20	− 2.00 ± 4.15	28	− 2.98 ± 3.37	0.3704
Ap	46	− 0.96 ± 1.36	19	− 1.16 ± 1.54	27	− 0.81 ± 1.23	0.4047
Bp	51	− 0.37 ± 1.27	23	− 0.07 ± 1.45	28	− 0.63 ± 1.06	0.1177
GH	41	4.23 ± 1.64	15	4.37 ± 0.64	26	4.15 ± 2.02	0.6232
PB	41	3.56 ± 0.56	15	3.50 ± 0.68	26	3.60 ± 0.49	0.6035
TVL	41	10.48 ± 4.79	15	9.67 ± 0.59	26	10.94 ± 5.99	0.2918
D	33	− 5.25 ± 2.47	13	− 5.35 ± 2.61	20	− 5.35 ± 2.61	0.8425
CMG/UDS, mean ± SD							
First sensation (m;)	151	181.68 ± 96.42	75	167.23 ± 92.09	76	195.95 ± 99.05	0.0671
Strong desire (ml)	151	303.34 ± 98.81	75	291.85 ± 92.75	76	314.68 ± 103.81	0.1564
Capacity (ml)	150	345.85 ± 93.54	74	330.05 ± 83.50	76	361.22 ± 100.56	0.0409
Volume (ml)	17	271.65 ± 99.65	10	265.00 ± 105.16	7	281.14 ± 98.55	0.7539
DO pressure (cmH_2_0)	17	11.00 ± 6.37	9	9.00 ± 3.71	8	13.25 ± 8.14	0.2059
SUI volume (ml)	132	277.22 ± 114.02	67	260.81 ± 103.31	65	294.14 ± 122.60	0.0932
MUCP (cmH_2_0)	154	74.18 ± 28.87	76	71.43 ± 28.56	78	76.86 ± 29.10	0.2450
PVR (ml)	54	16.04 ± 27.75	25	13.64 ± 24.63	29	18.10 ± 30.46	0.5606

*POPDI* Pelvic Organ Prolapse Distress Inventory, *DO* detrusor overactivity, *SUI* stress urinary incontinence, *MUCP* maximum urethral closure pressure, *PVR* post-void residual

Intra- and postoperative variables are presented in Table 3. Eighty-eight patients (57.8%) underwent MUS placement without concomitant prolapse surgery. The most commonly reported concomitant procedures were a combined anterior and posterior colporrhaphy (32.3%) followed by laparoscopic/robotic sacrocolpopexy (19.3%). Regarding the primary outcome, there was no difference in diagnosis of POUR between tamsulosin and placebo groups (17.7% vs 19.8%, *p* = 0.7420; Table 4). Furthermore, there were no differences in unanticipated health care encounters or complications within 30 days of surgery between the two groups (Table 4).

**Table 3:** Intraoperative factors

	Total (*N* = 161)		Placebo (*N* = 81)		Tamsulosin (*N* = 80)		*p* value
Type of anesthesia, *n* (%)							
MAC	92 (57.50)		49 (61.25)		43 (53.75)		0.3373
General	68 (42.50)		31 (38.75)		37 (46.25)		
ASA grade, *n* (%)							
1	21 (13.13)		10 (12.50)		11 (13.75)		0.7775
2	110 (68.75)		57 (71.25)		53 (66.25)		
3	29 (18.13)		13 (16.25)		16 (20.00)		
RMUS placement, *n* (%)							
No	161 (100.00)		81 (100.00)		80 (100.00)		–
Yes	0 (0.00)		0 (0.00)		0 (0.00)		
Concomitant prolapse surgery, *n* (%)							
None	93 (57.76)		51 (62.9)6		42 (52.50)		0.2754
Anterior repair	1 (0.62)		0 (0.00)		1 (1.25)		
Posterior repair/perineorrhaphy	15 (9.32)		5 (6.17)		10 (12.50)		
Combined AP repair	52 (32.30)		25 (30.86)		27 (33.75)		
Concomitant apical suspension, *n* (%)							
None	108 (67.08)		56 (69.14)		52 (65.00)		0.9172
Uterosacral vaginal vault suspension	10 (6.21)		5 (6.17)		5 (6.25)		
Sacrospinous vaginal vault suspension	12 (7.45)		5 (6.17)		7 (8.75)		
Laparoscopic/robotic sacrocolpopexy	31 (19.25)		15 (18.52)		16 (20.00)		
Concomitant procedure, *n* (%)							
None	114 (70.81)		60 (74.07)		54 (67.50)		0.2750
Vaginal hysterectomy	14 (8.70)		4 (4.94)		10 (12.50)		
Laparoscopic/robotics-assisted hysterectomy	27 (16.77)		15 (18.52)		12 (15.00)		
Colpocleisis	6 (3.73)		2 (2.47)		4 (5.00)		
Intraoperative complications, *n* (%)							
None	160 (99.38)		81 (100.00)		79 (98.75)		0.4969
Hemorrhage (EBL > 200 ml)	1 (0.62)		0 (0.00)		1 (1.25)		
EBL (ml), mean ± SD	142	49.71 ± 97.94	73	42.23 ± 54.77	69	57.62 ± 128.81	0.3615
Duration of procedure (min), mean ± SD	157	77.01 ± 83.83	80	71.54 ± 85.39	77	82.69 ± 82.34	0.4065

*ASA* American Society of Anesthesiologists, *RUMS* retropubic mid-urethral sling, *EBL* estimated blood loss

**Table 4:** Postoperative factors

	Total (*N* = 161)		Placebo (*N* = 81)		Tamsulosin (*N* = 80)		*p* value
Postoperative pain score on discharge (0–10), mean ± SD	154	2.31 ± 1.86	77	2.14 ± 1.80	77	2.48 ± 1.91	0.2603
Postoperative admission, *n* (%)							
No	137 (85.09)		70 (86.42)		67 (83.75)		0.6344
Yes	24 (14.91)		11 (13.58)		13 (16.25)		
Postoperative urinary retention, *n* (%)							
No	130 (81.25)		65 (80.25)		65 (82.28)		0.7420
Yes	30 (18.75)		16 (19.75)		14 (17.72)		
Unanticipated health care encounters (30 days), *n* (%)							
None	151 (93.79)		77 (95.06)		74 (92.50)		0.4609
UG office visit	2 (1.24)		1 (1.23)		1 (1.25)		
ED visit	2 (1.24)		0 (0.00)		2 (2.50)		
Readmission	3 (1.86)		2 (2.47)		1 (1.25)		
Unplanned phone call	2 (1.24)		0 (0.00)		2 (2.50)		
Patient portal message	1 (0.62)		1 (1.23)		0 (0.00)		
Complications 30 days after surgery, *n* (%)							
None	156 (96.89)		80 (98.77)		76 (95.00)		0.3680
UTI	2 (1.24)		1 (1.23)		1 (1.25)		
Reoperation	1 (0.62)		0 (0.00)		1 (1.25)		
Syncope	2 (1.24)		0 (0.00)		2 (2.50)		

*UG* urogynecology, *ED* Emergency Department, *UTI* urinary-tract infection

A subgroup analysis was performed focusing on patients who had MUS without concomitant surgery. Sixty patients in the placebo group versus 54 in the tamsulosin group met this criterion. Within this subgroup, POUR was lower within the tamsulosin group (7.5%) than in the placebo group (16.7%); however, this difference did not reach statistical significance (*p* = 0.142).

## Discussion

In this study a single dose of preoperative tamsulosin did not reduce the rate of POUR in patients undergoing MUS surgery. Although this regimen of tamsulosin did not increase complications and unanticipated health care encounters 30 days after surgery, it did not have a beneficial effect on our patient population. Subgroup analysis of patients who underwent MUS only without concomitant surgery revealed a trend toward lower POUR rates in the tamsulosin group; however, this difference was not statistically significant. The retro-fill voiding trial is a commonly used diagnostic method for POUR and is widely utilized in clinical practice [15, 16].

The mechanism of POUR following MUS placement is fundamentally different from that in other surgeries, as the iatrogenic urethral obstruction created by the sling is primarily mechanical rather than due to functional bladder-neck obstruction [18–20]. Given that tamsulosin’s primary effect is on smooth muscle relaxation in the prostate and urethra, its ability to counteract mechanical obstruction from the sling is likely limited [21]. This distinction may explain why no significant reduction in POUR was observed in our study. Although our subgroup analysis suggested a potential trend toward lower POUR rates in MUS-only patients, this was not statistically significant, and further investigation into urethral hypermobility and intrinsic sphincter dysfunction would be necessary to establish a biologically plausible mechanism for any potential benefit. Nevertheless, our findings remain clinically significant, particularly in health care settings where prophylactic tamsulosin administration is common despite limited evidence. By demonstrating that a single preoperative dose clearly does not prevent POUR, this study provides important guidance for clinical decision-making and challenges the routine, empirical use of tamsulosin in this context.

Many women consider being discharged home with a Foley catheter to be a surgical complication and describe catheter use as the worst aspect of their surgery [4, 22]. Indwelling catheters are the leading cause of hospital-acquired urinary-tract infections (UTIs), are often a source of embarrassment and inconvenience for patients, and may require additional office visits and health care utilization [23]. Continued research should be aimed at evaluating methods of reducing POUR in our patients to improve outcomes and patient satisfaction after surgery.

To our knowledge, this is the first study to evaluate whether a single dose of tamsulosin may impact POUR following MUS in a double-blind randomized controlled trial setting. Previous studies focusing on the impact of alpha-adrenergic receptor blockers have shown promising results. Livne et al. published a study evaluating postoperative a urinary retention decrease of 79.2% after postoperative administration of dibenzyline (an alpha-adrenergic receptor blocker) in women undergoing hysterectomy (postoperative urinary retention rate of 18.75% in controls versus 3.9% in the treatment group) [9]. Additional studies have also been published evaluating postoperative urinary retention in men and women undergoing various surgeries and have demonstrated a decrease in postoperative urinary retention after tamsulosin administration from 72 to 88% compared with controls [7, 10, 11]. These studies vary in tamsulosin administration regimen from multiple days preoperatively and postoperatively to a single postoperative dose; however, no studies have been published evaluating a single preoperative dose of tamsulosin and the effect on POUR [24, 25]. In contrast to these findings, the current study found no advantage in a single preoperative dose of tamsulosin in the context of POUR. This difference may be explained by variations in treatment regimens between studies as well as varying rates of POUR within different patient populations.

Clinically speaking, results of the current study do not support treatment with preoperative tamsulosin in patients undergoing MUS. Although tamsulosin reaches a steady state in approximately 5 days, it can reach the maximum blood concentration in approximately 4–5 h [10, 15]. This fact and the simplicity of a single preoperative dose led us to choose this regimen for our treatment arm. However, this study does not address the efficacy of other tamsulosin treatment protocols or that of other alpha-adrenergic receptor blockers. Also, we did not assess the impact of tamsulosin when administered on postoperative day (POD) 1. Future research utilizing different treatment protocols is needed in order to establish whether tamsulosin has any benefit in this clinical context. Data presented in this study include both patients undergoing MUS alone and those treated with concomitant prolapse repair. Although the current study was underpowered to evaluate the impact of tamsulosin in patients undergoing MUS only, a trend was noted in favor of the tamsulosin group regarding POUR. Urinary retention following pelvic-organ-prolapse repair may be multifactorial in nature with pain, swelling of tissues, and change in vaginal axis all possibly contributing to this phenomenon. We hypothesize that tamsulosin may have a beneficial effect in patients undergoing MUS alone, which may be diluted when including patients with concomitant prolapse repair. Future studies interested in focusing on this subgroup will have to consider the lower POUR rate following MUS surgery without concomitant procedures, leading to the need for a larger study group. Strengths of this study include its RCT double-blinded design. The clear study protocol and treatment regimen make these results highly reproducible.

Limitations of this study include a limited sample size. Furthermore, we were unable to assess the impact of tamsulosin when administered on POD1, because within our clinical routine, the majority of patients are discharged on the day of surgery.

The study's main strengths include its novel examination of a single preoperative dose of tamsulosin to prevent POUR in MUS surgeries. The randomized, double-blinded design and appropriate inclusion criteria enhance its validity. All surgeries were performed using the same procedure and implants with a similar technique, and the clear study protocol and treatment regimen make the results highly reproducible. However, limitations include the heterogeneity introduced by including patients both with and without concomitant prolapse repairs. This study also had uncontrolled variables such as differing anesthesia types, which further constrain the findings. The limited sample size hindered the ability to focus on subgroups within the patient population, and the inability to assess the impact of tamsulosin administered on POD1—because most patients were discharged on the day of surgery—also limited the study. These factors suggest that although the study provides valuable insights, its conclusions should be interpreted with caution. Future studies with larger, more MUS-focused patient populations without any concomitant procedures and better postoperative and post-discharge follow up are needed to assess the outcomes.

The study's main strengths include its novel examination of a single preoperative dose of tamsulosin to prevent POUR in MUS surgeries. To our knowledge, this study is the first randomized, double-blinded, placebo-controlled trial to evaluate the effect of a single preoperative dose of tamsulosin on POUR in MUS surgery. The study design enhances its validity by reducing bias and ensuring robust comparisons. Additionally, all procedures were performed using the same MUS product and surgical technique, improving reproducibility. The clear study protocol and treatment regimen further support the generalizability of these findings.

One limitation is the heterogeneity of the study population, as both patients undergoing MUS alone and those undergoing concomitant prolapse repair were included. This may have diluted any potential effect of tamsulosin in reducing POUR. Additionally, the study was not powered to detect differences in the MUS-only subgroup, despite a trend suggesting a potential benefit in this population. Another limitation is the lack of alternative tamsulosin dosing protocols, such as multi-day preoperative or postoperative administration, which may yield different results. Furthermore, variations in anesthesia techniques were not controlled for, which could have influenced urinary-retention rates. Finally, because most patients were discharged on the same day as surgery, the impact of tamsulosin on POUR beyond the immediate postoperative period remains unclear.

In conclusion, a single dose of tamsulosin prior to MUS surgery does not appear to decrease the POUR rate. Future research should focus on different tamsulosin treatment protocols as well as specific surgical procedures in which preoperative tamsulosin may have a positive effect.

## Data Availability

Due to concerns for privacy, the data underlying the results presented in the study are not publicly available. Data are available from the corresponding author upon reasonable request.
